# Noninvasive MGMT-promotor methylation prediction in high grade gliomas using conventional MRI and deep learning-based segmentations

**DOI:** 10.3389/fnins.2025.1689003

**Published:** 2025-12-16

**Authors:** Edin Zahirovic, Tim Salomonsson, Malte Knutsson, Xavier Saenz Sarda, Jimmy Lätt, Sara Kinhult, Mattias Belting, Anna Rydelius, Johan Bengzon, Linda Knutsson, Pia C. Sundgren

**Affiliations:** 1 Division of Radiology, Department of Clinical Sciences, Skåne University Hospital, Lund University, Lund, Sweden; 2 Division of Pathology, Department of Clinical Sciences, Skåne University Hospital, Lund University, Lund, Sweden; 3 Department of Medical Imaging and Physiology, Skåne University Hospital, Lund, Sweden; 4 Department of Clinical Sciences, Division of Oncology, Lund University, Lund, Sweden; 5 Division of Neurology, Department of Clinical Sciences, Skåne University Hospital, Lund University, Lund, Sweden; 6 Kamprad Laboratory, Division of Neurosurgery, Department of Clinical Sciences, Skåne University Hospital, Lund University, Lund, Sweden; 7 F. M. Kirby Research Center for Functional Brain Imaging, Kennedy Krieger Institute, Baltimore, MD, United States; 8 Department of Neurology, Johns Hopkins University School of Medicine, Baltimore, MD, United States; 9 Department of Medical Radiation Physics, Lund University, Lund, Sweden; 10 Lund University Bioimaging Center (LBIC), Lund University, Lund, Sweden

**Keywords:** MGMT, methylation, MRI, deep learning, high grade glioma (HGG)

## Abstract

**Background/objectives:**

High grade gliomas (HGG) are aggressive brain tumors, most frequently glioblastoma and astrocytoma grade 4. Methylation of O6-methylguanine-DNA methyltransferase (MGMT) promoter in HGG is crucial for temozolomide efficacy. As MGMT promoter methylation (MGMTpm) assessment requires tumor tissue, magnetic resonance imaging (MRI) is of interest for non-invasive prediction. We aimed to analyze volumetric data from edema, contrast-enhancing tumor, necrosis, total-tumor and total-tumor/edema ratio for MGMTpm prediction in HGG. Further we assessed overall survival (OS) and progression free survival (PFS) between groups and volumes.

**Methods:**

Segmentation was performed using deep learning models (DL-models), DeepBraTumIA and Raidionics, on 70 HGG patients (45 males, 32 MGMTpm). Manual segmentation was conducted in 37 for validation of DL-models. Group differences were evaluated using Man-Whitney U tests and receiver operation characteristic (ROC) curves. Multivariate analysis was conducted using logistic regression and bootstrapping. Dice coefficient, intraclass correlation coefficient (ICC) and Kruskal–Wallis test evaluated DL-model performance.

**Results:**

MGMTpm tumors displayed significantly larger edema, segmented by DeepBraTumIA (*p* = 0.03), and lower total-tumor/edema ratio segmented by both DL-models (*p* < 0.01). Raidionics segmented total-tumor/edema ratio showed highest univariate predictive ability with area under curve 0.687 (sensitivity 46.2%, specificity 87.5%). Multivariate analysis confirmed this, showing that the ratios from both DL-models were the only ROIs to remain independent, significant predictors (*p* < 0.05) after controlling for clinical covariates. The overall multivariate models were significant (*p* = 0.01) and improved prediction over baseline. ICC showed interclass correlation of 0.96 (contrast-enhancing tumor), 0.50 (tumor necrosis) and 0.90 (peritumoral edema). Segmentation methods demonstrated 83–91% median overlap in contrast-enhancing tumor, 67–80% in necrosis and 80–84% in edema regions. Significant OS and PFS differences were observed, notably being longer in MGMTpm tumors and lower tumor volumes.

**Conclusion:**

This study suggests that significant radiological differences in MGMTpm can be found using deep learning models, primarily in tumor edema volume. MGMTpm status and region of interest volumes impact OS and PFS. Future studies should incorporate other molecular imaging sequences for methylation prediction.

## 1 Introduction

Grade 4 glioblastoma (GBM) and astrocytoma grade 4 are highly aggressive tumors, both being high grade gliomas (HGG) (Ostrom et al., 2018; Louis et al., 2021). The distinction between these two gliomas is based on isocitrate dehydrogenase (IDH) mutation status (Torp et al., 2022). Astrocytoma expresses an IDH mutation, while GBM is IDH-wildtype (Torp et al., 2022). Despite modern treatment, both tumor entities have poor survival rate (Yan et al., 2009).

Standard of care for newly diagnosed GBM and astrocytoma consists of surgical resection followed by radiotherapy (RT) with concomitant temozolomide (TMZ), followed by adjuvant TMZ according to the Stupp protocol (Stupp et al., 2005; Witthayanuwat et al., 2018; Fernandes et al., 2017). However, the combined treatment regimen has shown considerable variances in tumor response, especially regarding TMZ (Hegi et al., 2005; Hegi et al., 2024).

It has been established that this is due to the methylation of the O6-methylguanine-DNA methyltransferase (MGMT) promoter (Hegi et al., 2005; Hegi et al., 2024; Singh et al., 2021). MGMT plays a crucial role in modulating cell survival through repairing DNA damage by removing alkyl groups from the O6 position of guanine (Singh et al., 2021). Since TMZ exerts its cytotoxic effect by adding an alkyl group to this position (Hegi et al., 2024), an active MGMT enzyme can counteract TMZ, rendering it ineffective (Hegi et al., 2024). Methylation of the MGMT-promoter limits MGMT production within the cell (Esteller and Herman, 2004).

Testing for MGMT methylation is done through DNA sequencing methods such as pyrosequencing or methylation specific polymerase chain reaction (PCR) on tumor tissue (Hegi et al., 2005; Wick et al., 2012). Furthermore, MGMT testing is rarely done in HGG recurrence, despite tumor heterogeneity and potential variance in MGMT methylation (Weller et al., 2013; Mansouri et al., 2019). Thus, there is a need for noninvasive, dynamic testing of MGMT promoter methylation in newly diagnosed HGG. Magnetic resonance imaging (MRI) provides a noninvasive approach that accounts for tumor heterogeneity (Suh et al., 2018).

There are some previous studies that, with different methods, have explored the use of MRI for predicting MGMT promoter methylation in HGG. A meta-analysis found that MGMT-methylated tumors typically exhibit less aggressive MRI features on conventional MRI, such as reduced peritumoral edema and tumor burden, compared to non-methylated (Suh et al., 2018). Other studies also found predictive potential in peritumoral edema volume (Ellingson et al., 2012; Kanas et al., 2017). Additionally, other studies found no predictive ability of MGMT promoter methylation in peritumoral edema (Carrillo et al., 2012; Kim et al., 2022), but it is concluded that the edema volume could impact survival if the tumor is MGMT methylated (Carrillo et al., 2012).

It is valuable for the neurosurgeon and the treating physician to have pre-operative knowledge about the MGMT status as it might influence pre-operative decision making. Therefore, using MRI for volumetric tumor characterization might aid in this distinction and could potentially be more effectively done using deep learning (DL) based models (Bonada et al., 2024).

The aim of this study is to evaluate imaging features on conventional MRI for MGMT promoter methylation such as tumor characteristics; tumor volume, tumor necrosis volume, and edema volume by using two different DL models, DeepBraTumIA (DeepBraTumIA, 2025) and Raidionics (Bouget et al., 2023; GitHub, 2023), to assess the DL-models ability to prediction of MGMT methylation using raw segmentation outputs. Thus, the novelty of our work lies in the real-world application of readily available DL-models with assessable user interfaces in predicting MGMTpm methylation. We will also assess overall survival (OS) and progression free survival (PFS) of MGMT promotor methylated vs. non MGMT promoter methylated tumors and higher vs. lower volumes of regions of interest (ROIs).

## 2 Materials and methods

### 2.1 Patient recruitment

In this retrospective study 70 patients diagnosed with HGG [GBM grade 4 and astrocytoma grade 4 according to the WHO 2021 classification (Louis et al., 2021)] were included from a larger cohort of an ongoing project at the Department of Clinical Sciences, Division of Diagnostic Radiology, Lund University. The study was conducted in accordance with the Declaration of Helsinki, and approved by the Regional Ethics Board Lund, Sweden and by the Swedish Ethical Review Authority prior to starting any work. Informed consent was obtained from all subjects involved in the study. The inclusion criteria were as follows: >18 years old, undergone biopsy or resection of the brain lesion for pathological analysis, and completion of preoperative MRI examination, including conventional MRI sequences. Exclusion criteria were having a pacemaker or metallic wires in the body incompatible with MRI and being unable to sign informed consent.

### 2.2 MGMT analyses

MGMT status was routinely assessed using pyrosequencing, performed at the Division of Pathology, Skåne University Hospital, Lund, Sweden (Mikeska et al., 2007). A cut-off ≥10% was defined as MGMT promoter methylation.

### 2.3 Image acquisition

Thirty-nine patients were examined on a MAGNETOM Prisma 3T MRI scanner (Siemens Healthcare, Erlangen, Germany) equipped with a 20-channel head coil. Whole-brain imaging sequences included: T2 Turbo spin echo with a repetition time (TR) = 6,000 ms, echo time (TE) = 100 ms, in-plane resolution = 1 × 1 mm^2^, slice thickness = 5 mm; T2-weighted Fluid-Attenuated Inversion Recovery (T2-FLAIR) with a TR = 5,000 ms, inversion time (TI) = 1800 ms, TE = 393 ms, in-plane resolution = 1 × 1 mm^2^, slice thickness = 1 mm, and pre- and post-gadolinium contrast T1 (T1-GD) magnetization prepared rapid gradient echo (MPRAGE) with a TR = 1900 ms, TI = 900 ms, TE = 2.54 ms, in-plane resolution = 1 × 1 mm^2^, slice thickness = 1 mm.

Thirty-one patients were examined on a MAGNETOM Skyra 3T system (Siemens Healthcare, Erlangen, Germany). The MRI protocol consisted of morphological imaging; T2 Turbo spin echo (TE/TR = 100 ms/6,870 ms), T2-FLAIR (TE/TR/TI = 81 ms/9,000 ms/2,500 ms) and T1-MPRAGE (TE/TR/TI = 2.54 ms/1,900 ms/900 ms, 1 mm isotropic voxels).

### 2.4 Measurements

**Figure 1:**
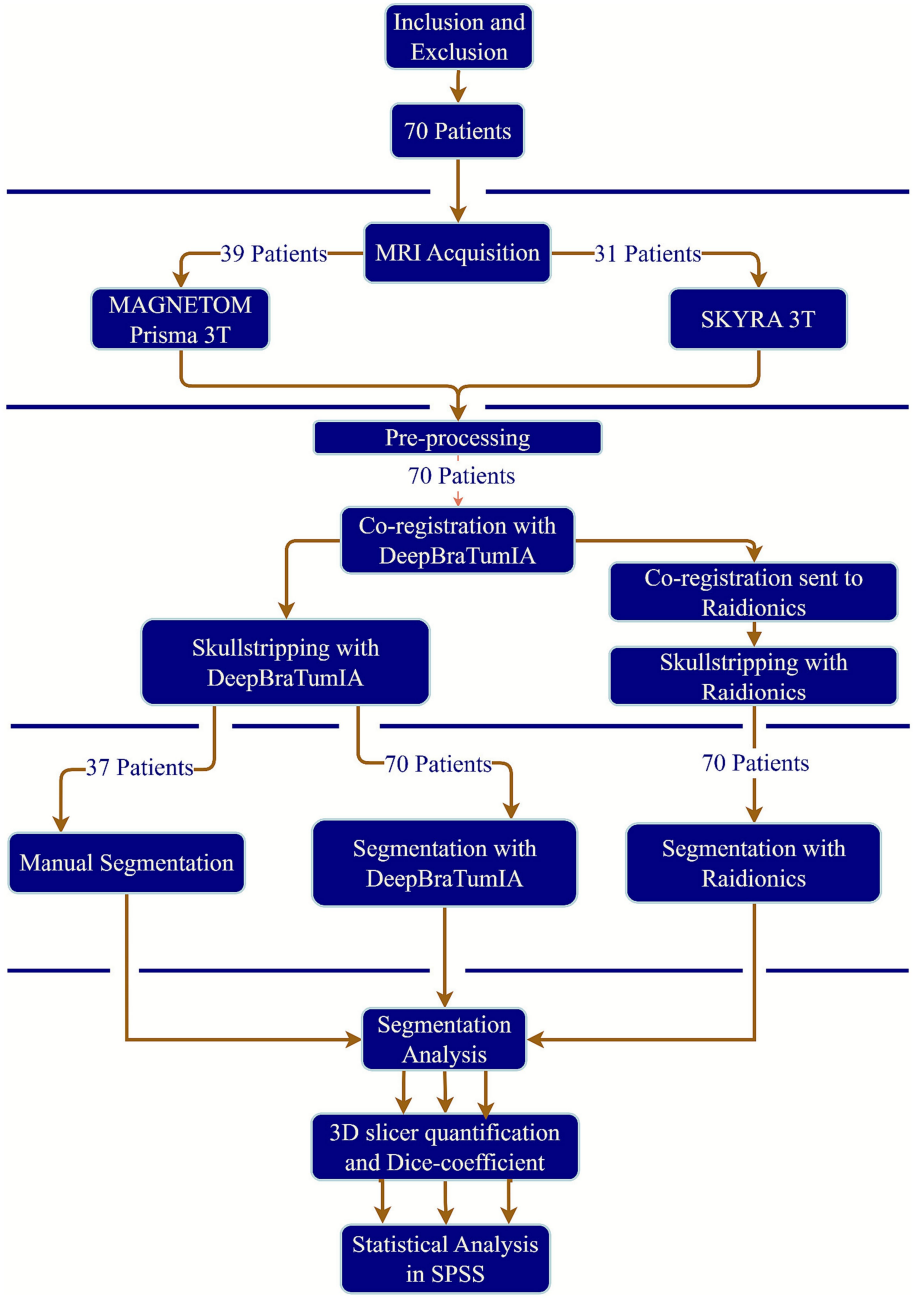
Workflow diagram summarizing the study pipeline, from patient inclusion (*n* = 70) through image acquisition, pre-processing, segmentation, and final analysis.

Three separate primary segmentations (manual- and two DL-model segmentations) were performed on the tumors to determine peritumoral edema, tumor necrosis, and contrast-enhancing tumor volumes. As part of the processing pipeline in DeepBraTumIA (DeepBraTumIA, 2025), the images were co-registered to the MNI152 atlas with a rigid transform, 1 mm isotropic voxel size and matrix size 182 × 218 × 182, using the T1-GD scan as reference. Skull-stripping was then performed using the HD Brain Extraction Tool (HD-BET) (Isensee et al., 2019). Manual segmentations were conducted on the registered, skull-stripped images while Raidionics (Bouget et al., 2023) was done on registered images. Voxel-based volume measurements were performed in 3D Slicer (slicer.org, version 5.6.2) using the Quantification of Segmentation module, where one isotropic voxel within the ROI corresponded to one cubic millimeter (Fedorov et al., 2012). Dice-coefficients were calculated using segment comparison module from SlicerRT (version 7168e01) extension in 3D slicer (Pinter et al., 2012). A visual workflow summarization from MRI acquisition to DL-segmentation and final analysis can be seen in Figure 1.

### 2.5 DL-segmentation

Both DeepBraTumIA and Raidionics are comprehensive, ready-to-use software packages that require no additional training or preprocessing on the end-user’s data (DeepBraTumIA, 2025; Bouget et al., 2023). DeepBraTumIA employs a modified U-net architecture, so called no-new-netU-net (nnU-net) for all segmentations tasks (DeepBraTumIA, 2025). NnU-Nets have shown promising results in other segmentation tasks such as post-operative segmentation of GBM (Cepeda et al., 2024). In contrast, Raidionics utilizes an attention-gated U-net (AGU-net) architecture (Bouget et al., 2023). Both models are trained on pre- and post-operative brain tumors (DeepBraTumIA, 2025; Bouget et al., 2023). However, it should be noted that no quality assessment or post processing of individual segmentation from the DL-models was conducted in this study.

### 2.6 Manual segmentation

For manual segmentation, registered and skull-stripped images were imported into 3D Slicer. Manual segmentation was performed on 37 out of 70 subjects to serve as a validation benchmark for the DL models. Using T1-GD, a ROI for the contrast-enhancing tumor was identified as the hyperintense region relative to the surrounding tissue, excluding any vessels. The tumor necrosis ROI was measured using T1-GD and defined as a hypointense area in T1-GD within the tumor ROI. Lastly, the edema ROI was identified as the hyperintense area on FLAIR images excluding the contrast-enhanced area and necrosis identified using the T1-GD. Manual segmentation was performed by a junior researcher (EZ), under the guidance of a junior consultant (TS) and a board certified neuroradiologist (PS). Individual segmentations were sampled and quality assessed by TS and PCS. During the segmentation process, the author (EZ) was blinded to MGMT-status and to the results of the other segmentation models.

### 2.7 Statistical analysis

All statistical analyses were performed using IBM SPSS (version 29.0.2.0). The ROIs analyzed were tumor volume, tumor necrosis volume, peritumoral edema volume, total tumor volume consisting of the sum of tumor volume, tumor necrosis volume, and the total-tumor/edema ratio.

Differences in betamethasone (corticosteroid) usage between MGMT groups were assessed using a Mann–Whitney *U* test while remaining patient characteristics were compared using chi-square tests. To assess differences in ROIs between non-methylated and methylated MGMT promoter tumors the Mann–Whitney *U* test was applied. ROIs from DeepBraTumIA and Raidionics were separately tested. The ROIs’ volumes predictive ability were assessed using receiver operating characteristic (ROC) curves, also calculating the Youden optimal cutoff. For multivariate testing a logistic regression with bootstrapping (1,000 samples) was performed with an individual ROI, age, sex, tumor type, corticosteroid dose and IDH as covariates. Logistic regression was also performed excluding IDH status due to small sample size causing large confidence intervals, model instability and lack of confidence in result interpretation.

For comparisons between the volumetric data from volumes of manual ROIs and those generated by DL models, a Wilcoxon signed ranks test was used. Statistical significance was defined as *p* < 0.05. Furthermore, inter-observer reliability among segmentation methods was evaluated using intraclass correlation coefficient (ICC).

Survival analysis of OS and PFS was performed using the Kaplan–Meier method and log-rank test, comparing MGMT-promoter methylated and unmethylated tumors. For each ROIs’ volume the same approach was used to compare OS and PFS between tumors with volumes above the median, defined as higher volume, and those with volumes at or below the median, defined as lower volume.

## 3 Results

### 3.1 Patient characteristics

A total of 70 patients (45 males, 25 females; mean age: 60 years) with histologically confirmed HGG (60 GBM and 10 astrocytoma WHO grade 4) and known MGMT promoter methylation status were included. Among these, 32 tumors had a methylated MGMT promoter, while the remaining 38 were unmethylated. Five of the tumors did not have histologically determined IDH status due to lacking sufficient material for the analysis. These tumors were classified as GBM, using the histological morphology and other pathology markers, by a senior board-certified pathologist (XS). For patient demographics and tumor characteristics see Table 1.

**Table 1:** Patient characteristics, including demographic, histological tumor classification and tumor localization.

Variables	MGMT Met	Non MGMT met	All patients
Total patients	32 (100%)	38 (100%)	70 (100%)
Age at scan date (years), mean	57.8	62.5	60.3
Gender, *n* (%)			
Male	20 (63%)	25 (64%)	45 (64%)
Female	12 (37%)	13 (36%)	25 (36%)
Scanner			
Prisma 3T	12 (38%)	19 (50%)	39 (56%)
Skyra 3T	20 (63%)	19 (50%)	31 (44%)
Tumor type, *n* (%)^a^			
GBM (including IDH unknown)	25 (78%)	36 (95%)	61 (87%)
Astrocytoma 4	7 (22%)	2 (5%)	9 (13%)
IDH status, *n* (%)			
Wildtype	24 (75%)	34 (89%)	58 (83%)
Mutated	6 (19%)	1 (3%)	7 (10%)
Missing data	2 (6%)	3 (8%)	5 (7%)
IDH 1 status, *n* (%)			
Wildtype	22 (69%)	30 (79%)	52 (74%)
Mutated	6 (19%)	1 (3%)	7 (10%)
Missing data	4 (13%)	7 (18%)	11 (16%)
IDH 2 status, *n* (%)			
Wildtype	21 (66%)	24 (63%)	45 (64%)
Mutated	1 (3%)	1 (3%)	2 (3%)
Missing data	10 (31%)	13 (34%)	23 (33%)
Tumor location, *n* (%)			
Frontal lobe	9 (28%)	12 (32%)	21 (30%)
Parietal lobe	9 (28%)	9 (24%)	18 (26%)
Temporal lobe	5 (16%)	12 (32%)	17 (24%)
Gyrus cingulate	5 (16%)	2 (5%)	7 (10%)
Occipital lobe	1 (3%)	2 (5%)	3 (4%)
Cerebellum	1 (3%)	0 (0%)	1 (1%)
Insula	1 (3%)	0 (0%)	1 (1%)
Nucleus caudatus	0 (0%)	1 (3%)	1 (1%)
Tumor lateralization, *n* (%)			
Left hemisphere	15 (47%)	21 (54%)	36 (51%)
Right hemisphere	15 (47%)	13 (36%)	28 (40%)
Midline	2 (6%)	4 (10%)	6 (9%)
Betamethasone dosage (mg), mean	3.6	3.7	3.7

Due to rounding errors, columns may sum to >100%. Met., methylated; IDH, isocitrate dehydrogenase.

a Significant difference in distribution between O6-methylguanine-DNA methyltransferase (MGMT) and non-MGMT methylated group.

Significant differences in tumor type between the MGMT-methylated and non-methylated groups were observed (*p* = 0.02). The non-methylated group had a higher proportion of GBM [36 (95%) vs. 24 (75%)], while the methylated group had a comparatively higher proportion of grade 4 astrocytomas [8 (25%) vs. 2 (5%)] (Table 1). The remaining characteristics in Table 1 demonstrated no significant differences (data not shown).

### 3.2 Segmentation assessment

**Figure 2:**
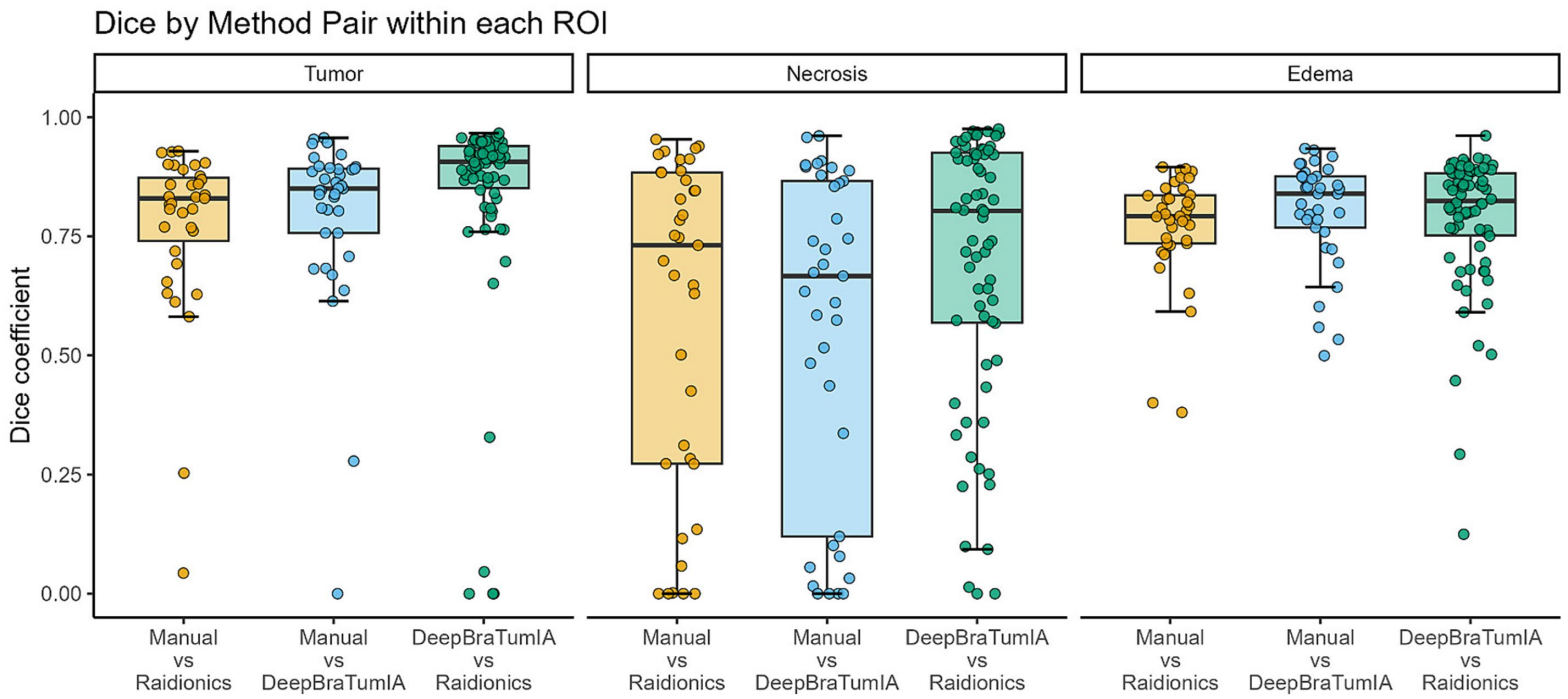
Box plot showing dice coefficient between segmentation methods within region of interest. Dice coefficients to manual from manually segmented images of 37 patients. Dice coefficients between deep learning (DL)-models are based on all 70 patients.

Comparing DeepBraTumIA to manual segmentation, significant differences were found in mean ranks in four out of five ROIs (tumor necrosis, total tumor volume, peritumoral edema and ratio) (Table 2). Compared to volumes from manual segmentation, both mean tumor necrosis and total tumor volume were significantly over segmented by 7,946 mm^3^ (*p* < 0.01) and 8,600 mm^3^ (*p* < 0.01) respectively (Table 2). Mean peritumoral edema was under segmented by 6,118 mm^3^ (*p* = 0.02) and ratio was overestimated (mean +0.144, *p* < 0.01) (Table 2). Comparing dice-coefficients across all 37 subjects shows a median overlap of 85.0% in contrast-enhancing tumor, 66.7% in tumor necrosis and 83.9% in peritumoral edema (Figure 2).

**Table 2:** Mean volumes of regions of interest (ROIs) from manually segmented images of 37 patients and deep learning (DL) models segmentation in the same images.

Segmentation method: Manual			
ROI	Mean volume (mm^3^)	ROI	Mean volume (mm^3^)
CE tumor	12,734	Tumor necrosis	5,405
Total tumor	18,139	Peritumoral edema	73,701
Total-tumor/edema ratio	0.294		

Segmentation method: DeepBraTumIA							
ROI	Mean volume (mm^3^)	Difference to manual segmentation	*p*-value	ROI	Mean volume (mm^3^)	Difference to manual segmentation	*p*-value
CE tumor	13,389	+655	*p* = 0.52	Tumor necrosis	13,351	+ 7,946	*p* < 0.01
Total tumor	26,740	+8,601	*p* < 0.01	Peritumoral edema	67,583	−6,118	*p* = 0.02
Total-tumor/edema ratio	0.439	+0.144	*p* < 0.01				

Segmentation method: Raidionics							
ROI	Mean volume (mm^3^)	Difference to manual segmentation	*p*-value	ROI	Mean volume (mm^3^)	Difference to manual segmentation	*p*-value
CE tumor	13,061	+327	*p* = 0.07	Tumor necrosis	6,572	+1,167	*p* = 0.51
Total tumor	19,633	+1,494	*p* = 0.01	Peritumoral edema	72,163	−1,539	*p* = 0.32
Total-tumor/edema ratio	0.324	+0.030	*p* = 0.21				

Each mean of DL segmented ROI is compared to the mean of the corresponding manually segmented ROI and displayed as a difference. *p*-value shows a significant level of difference, using Kruskal–Wallis, to corresponding ROI using manual segmentation. CE tumor, contrast enhancing tumor. A *p* < 0.05 indicates significant differences.

Comparing Raidionics to manual segmentation, significant overestimation of total tumor volume by 1,494 mm^3^ (*p* = 0.01) was observed (Table 2). No significant differences were observed for the other ROIs. Comparing dice-coefficients across all 37 subjects shows a median overlap of 83.0% in contrast-enhancing tumor, 73.1% in necrosis and 79.2% in edema (Figure 2).

Comparing segmentations between the two DL-models across all 70 subjects shows a median overlap of 90.7% in contrast-enhancing tumor, 80.3% in necrosis and 82.4% in edema (Figure 2).

**Figure 3:**
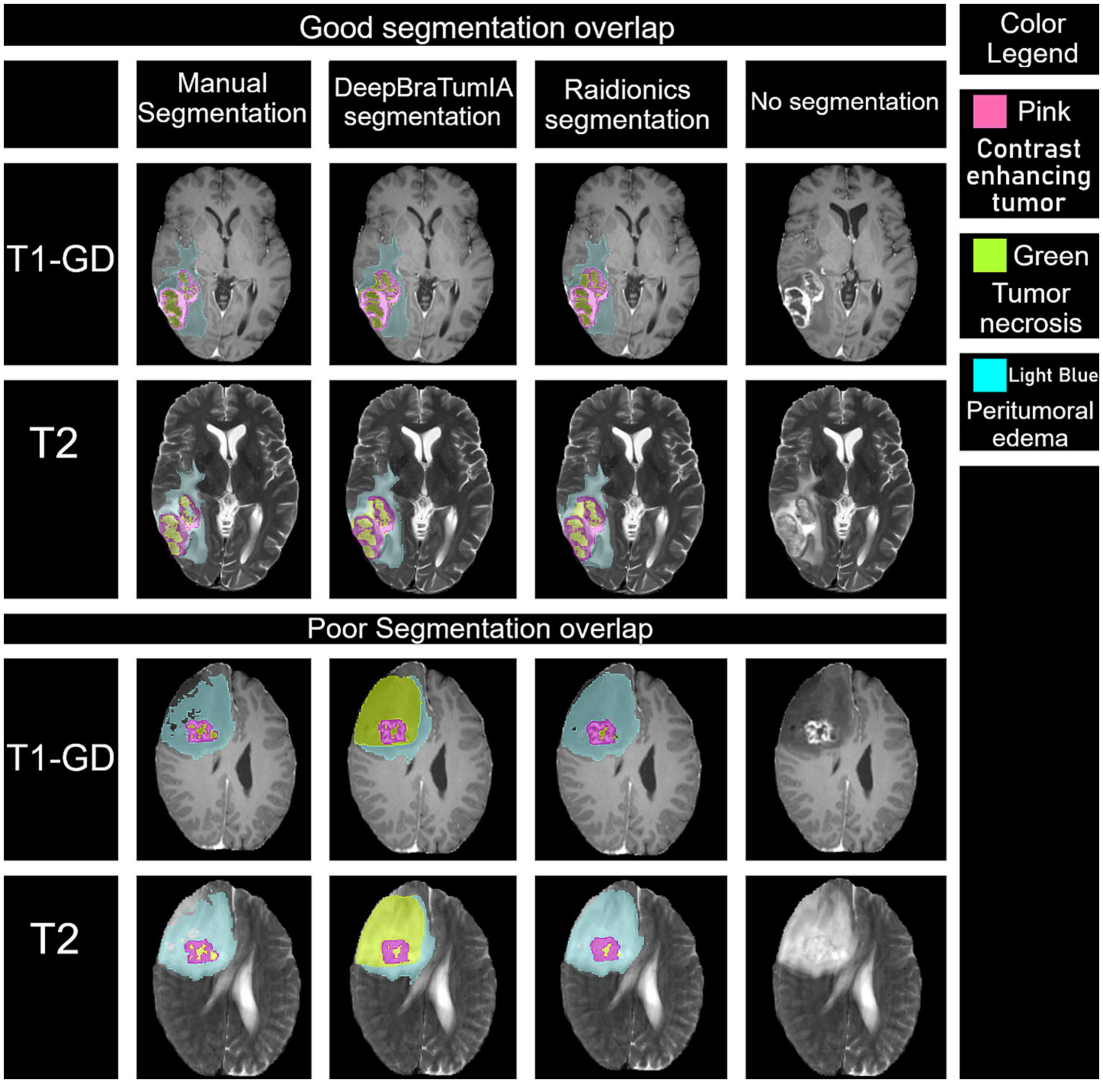
Depiction of the marked region of interest (ROIs) on the same slice in T1-GD and T2 images between manual, DeepBraTumIA and Raidionics segmentation. Pink is contrast enhancing tumor ROI, green is tumor necrosis ROI and light blue is peritumoral edema ROI. The image shows good and poor segmentation overlap. The good segmentation overlap showcases high agreement across all three measured ROIs and segmentation methods. The poor segmentation overlap showcases a case where DeepBraTumIA clearly mislabels an area with edema as necrosis.

Assessing all three segmentation methods using ICC showed interclass correlation of 0.96 (95% CI 0.94–0.98, *p* < 0.001) for the contrast-enhancing tumor, 0.50 (95% CI 0.14–0.73, *p* = 0.006) for tumor necrosis, and 0.90 (95% CI, 0.83–0.95, *p* < 0.001) for peritumoral edema. These results indicate excellent, poor to moderate and good to excellent reliability, respectively. Figure 3 shows a visual assessment of inter-observer agreement among the different segmentation methods. Two assessments are shown, one demonstrates good overlap in all segmented ROIs, and one reveals a poor overlap through mislabeling of the tumor necrosis ROI.

### 3.3 MGMT-prediction

#### 3.3.1 Mixed cohort

Significant differences between non methylated and methylated MGMT tumors were observed in the total tumor/edema ratio ROI in both DL-models (DeepBraTumIA *p* = 0.02, Raidionics *p* < 0.01), and in the edema volumes extracted from DeepBraTumIA (*p* < 0.05) (Table 3). Peritumoral edema was significantly larger in the methylated group (mean 81,934 mm^3^, Std 8,733 mm^3^) compared to non-methylated (mean 61,249 mm^3^, Std 6,471 mm^3^) (Table 3). The total tumor/edema ratio was significantly lower in MGMT methylated group from both DeepBraTumIA (0.417, Std 0.062) and Raidionics (0.332, Std 0.048) segmentations compared to non MGMT methylated [DeepBraTumIA (0.876, Std 0.213) and Raidionics (0.747, Std 0.120)] (Table 3).

**Table 3:** Mean volumes from each region of interest (ROI) segmented by both deep learning models separated by O6-methylguanine-DNA methyltransferase (MGMT) methylation status.

Segmentation method: DeepBraTumIA		
ROI	Sub-group	Volume (mm^3^)
CE tumor volume	MGMT met	18,006
	Non-met	23,277
	Difference	−5,271
	*p*-value	*p* = 0.20
Tumor necrosis volume	MGMT met	12,753
	Non-met	12,746
	Difference	+7
	*p*-value	*p* = 0.24
Total tumor volume	MGMT met	30,760
	Non-met	36,024
	Difference	−5,264
	*p*-value	*p* = 0.26
Peritumoral edema volume	MGMT met	81,934
	Non-met	61,249
	Difference	+20,685
	*p*-value	p < 0.05
Total-tumor/edema ratio	MGMT met	0.417
	Non-met	0.876
	Difference	−0.456
	*p*-value	*p* = 0.02

Segmentation method: Raidionics		
ROI	Sub-group	Volume (mm^3^)
CE tumor volume	MGMT met	19,861
	Non-met	23,750
	Difference	−3,889
	*p*-value	*p* = 0.27
Tumor necrosis volume	MGMT met	5,591
	Non-met	10,400
	Difference	−4,809
	*p*-value	*p* = 0.41
Total tumor volume	MGMT met	25,452
	Non-met	34,150
	Difference	−8,698
	*p*-value	*p* = 0.11
Peritumoral edema volume	MGMT met	85,848
	Non-met	64,184
	Difference	+21,664
	*p*-value	*p* = 0.10
Total-tumor/edema ratio	MGMT met	0.332
	Non-met	0.747
	Difference	−0.415
	*p*-value	*p* < 0.01

Differences are measured from the MGMT methylated group to the non-methylated group. A *p* < 0.05 is defined as significant and signifies a significant difference in volumes between methylated and non-methylated MGMT promoter. CE tumor, contrast enhancing tumor; Met., methylated.

**Figure 4:**
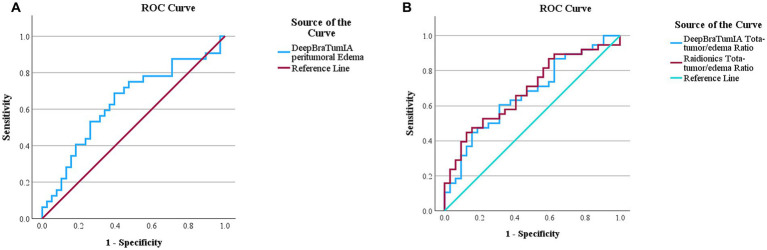
**(A)** Receiver operation characteristics (ROC) curve for O6-methylguanine-DNA methyltransferase (MGMT)-promoter methylation prediction using peritumoral edema region of interest segmented using DeepBraTumIA. AUC 0.638, 95% CI 0.504–0.772. **(B)** Receiver operation characteristics (ROC) curve for non O6-methylguanine-DNA methyltransferase (MGMT)-promoter methylation prediction using total-tumor/edema ratio segmented by DeepBraTumIA and Raidionics. DeepBraTumIA AUC 0.667, 95% CI 0.540–0.794, Raidionics AUC 0.687, 95% CI 0.562–0.811.

ROC analysis indicated potential MGMT promotor methylation prediction in the peritumoral edema ROI from DeepBraTumIA (AUC 0.638, 95% CI 0.504–0.772, sensitivity 68.8%, specificity 61.5%, at Youden index cutoff 62,633 mm^3^) (Table 4). Visualization of the ROC-curve for DeepBraTumIA segmented peritumoral edema can be seen in Figure 4A. The total tumor/edema ratio was predictive of non MGMT promotor methylation from DeepBraTumIA (AUC 0.667, 95% CI 0.540–0.794, sensitivity 60.5%, specificity 68.7%, at Youden optimal cutoff 0.449) and Raidionics (AUC 0.687, 95% CI 0.562–0.811, sensitivity 44.7%, specificity 87.5%, at Youden optimal cutoff 0.625) (Table 4). Visualization of the ROC-curve for DeepBraTumIA and Raidionics based total-tumor/edema ratio can be seen in Figure 4B.

**Table 4:** Area under curve (AUC) and Youden’s optimal cut off for all regions of interest (ROI’s) with significant results from receiver operation characteristics (ROC) analysis.

ROI	DeepBraTumIA peritumoral edema volume	DeepBraTumIA total-tumor/edema ratio	Raidionics total-tumor/edema ratio
AUC	0.64	0.67	0.69
Std	0.07	0.07	0.06
Asymptotic sig.	0.04	0.01	<0.01
95% CI	0.51–0.77	0.54–0.79	0.56–0.81
Cutoff	62,633	0.4489	0.6250
Sensitivity	0.688	0.605	0.447
Specificity	0.615	0.687	0.875
Youden’s index	0.29	0.29	0.32

Std, standard deviation; Sig., significance; 95% CI, 95% confidence interval.

Multivariate analysis was only performed on ROIs which were found to be significantly different in volume between MGMT methylated and non-methylated tumors. Initial modeling was performed using IDH as a covariate, however this proved to cause an unstable model, with large confidence intervals and skewed significance results as seen in Table 5. However, with and without IDH we observed similar results regarding ROI significance and effects of remaining covariates. When excluding IDH, two out of the three tested ROIs, resulted in overall statistically significant MGMT prediction model, those being ratio segmented by DeepBraTumIA (prediction improved to 67.1% from 54.3% (baseline), Omnibus test of model coefficients *p* = 0.01) and Raidionics (prediction improved to 65.7% from 54.3% (baseline), Omnibus test of model coefficients *p* = 0.01). More importantly, the ROIs were the only variables to stand out as independent, statistically significant predictors post bootstrapping with DeepBraTumIA ratio *p* = 0.03 (*B* = −2.34, 95% CI −7.63 to −1.03) and Raidionics ratio *p* = 0.02 (*B* = −2.09, 95% CI −6.49 to −1.02). Remaining co-variates were found to be non-significant (Table 5).

**Table 5:** Results of multivariate logistic regression with bootstrapping (1,000 samples) for prediction of O6-methylguanine-DNA methyltransferase (MGMT) methylation in high grade gliomas showing separate models for DeepBraTumIA ratio, DeepBraTum peritumoral edema and Raidionics ratio.

Logistical regression modeled with IDH status				
Segmentation method	Covariates	*B* (coefficient)	*p*-value	95% CI
DeepBraTumIA total-tumor/edema ratio	Age	−0.015	0.72	−0.19 to 0.06
	Sex	1.248	0.08	−0.07 to 4.30
	Tumor type	−1.142	0.17	−27.11 to 21.33
	Total-tumor/edema ratio	**−4.522**	**<0.01**	**−12.46 to** −**2.12**
	Corticosteroid dosage	0.023	0.90	−0.71 to 0.36
	IDH status	**5.83**	**0.01**	**−21.27 to 37.76**
DeepBraTumIA peritumoral edema volume (cm^2^)	Age	−0.004	0.89	−0.11 to 0.08
	Sex	0.807	0.22	−0.44 to 2.67
	Tumor type	−0.389	0.33	−24.03 to 22.10
	Peritumoral edema volume	0.008	0.29	−0.01 to −0.03
	Corticosteroid dosage	−0.98	0.50	−0.77 to 0.10
	IDH status	**2.883**	**0.03**	**−21.00 to 49.04**
Raidionics total-tumor/edema ratio	Age	−0.018	0.63	−0.16 to 0.06
	Sex	1.012	0.17	−0.27 to 3.51
	Tumor type	−1.098	0.20	−26.79 to 22.20
	Total-tumor/edema ratio	**−3.350**	**0.04**	**−10.10 to −1.00**
	Corticosteroid dosage	0.015	0.94	−0.67 to 0.30
	IDH status	**3.17**	**0.02**	**−21.38 to 50.18**

Logistical regression modeled without IDH status				
Segmentation method	Covariates	*B* (coefficient)	*p*-value	95% CI
DeepBraTumIA Total-tumor/edema ratio	Age	−0.044	0.09	−0.13 to 0.01
	Sex	0.297	0.65	−1.02 to 1.84
	Tumor type	1.572	0.13	−1.03 to 23.62
	Total-tumor/edema ratio	**−2.333**	**0.02**	**−6.50 to −0.99**
	Corticosteroid dosage	0.017	0.86	−0.21 to 0.29
DeepBraTumIA peritumoral edema volume (cm^2^)	Age	−0.023	0.41	−0.10 to 0.04
	Sex	0.210	0.69	−0.97 to 1.52
	Tumor type	1.223	0.16	−0.90 to 22.64
	Peritumoral edema volume	0.009	0.14	−0.01 to 0.03
	Corticosteroid dosage	−0.037	0.65	−0.30 to 0.13
Raidionics total-tumor/edema ratio	Age	−0.043	0.13	−0.14 to 0.01
	Sex	0.234	0.71	−1.16-1.70
	Tumor type	0.743	0.41	−2.10 to 21.46
	Total-tumor/edema ratio	**−2.107**	**0.01**	**−5.74 to −1.00**
	Corticosteroid dosage	0.010	0.90	−0.20 to 0.22

Models with and without isocitrate dehydrogenase (IDH) status as a covariate are shown. 95% CI, 95% confidence interval. Significant results are bold.

#### 3.3.2 GBM-subgroup

Significant differences between non methylated and methylated MGMT GBMs were observed in the total tumor/edema ratio ROI in both DL-models (DeepBraTumIA *p* < 0.01, Raidionics *p* < 0.01). The total tumor/edema ratio was significantly lower in MGMT methylated group from both DeepBraTumIA (0.338, Std 0.045) and Raidionics (0.335, Std 0.052) segmentations compared to non MGMT methylated [DeepBraTumIA (0.891, Std 0.225) and Raidionics (0.770, Std 0.125)].

**Figure 5:**
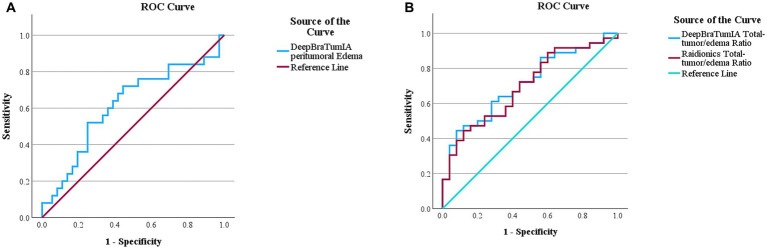
**(A)** Receiver operation characteristics (ROC) curve for O6-methylguanine-DNA methyltransferase (MGMT)-promoter methylation prediction in GBM subgroup using peritumoral edema region of interest segmented using DeepBraTumIA. 0.616, 95% CI 0.467–0.764. **(B)** Receiver operation characteristics (ROC) curve for non O6-methylguanine-DNA methyltransferase (MGMT)-promoter methylation prediction in GBM subgroup using total-tumor/edema ratio segmented by DeepBraTumIA and Raidionics. DeepBraTumIA AUC 0.711, 95% CI 0.582–0.840, Raidionics AUC 0.698, 95% CI 0.566–0.829.

ROC analysis showed best predictive ability of MGMT promotor methylation in peritumoral edema ROI from DeepBraTumIA (0.616, 95% CI 0.467–0.764, sensitivity 72.0%, specificity 55.6% at Youden optimal cutoff 53,076 mm^3^). Visualization of the ROC-curve for DeepBraTumIA segmented peritumoral edema can be seen in Figure 5A. Total tumor/edema ratio was predictive of non MGMT promotor methylation from DeepBraTumIA (AUC 0.711, 95% CI 0.582–0.840, sensitivity 44.4%, specificity 92.0%, at Youden optimal cutoff 0.625) and Raidionics (AUC 0.698, 95% CI 0.566–0.829, sensitivity 44.4%, specificity 88.0%, at Youden optimal cutoff 0.638). Visualization of the ROC-curve for DeepBraTumIA and Raidionics based total-tumor/edema ratio can be seen in Figure 5B.

### 3.4 Survival analysis

#### 3.4.1 Mixed cohort

OS and PFS were shown to be significantly higher (*p* < 0.01 and *p* = 0.03 respectively) in MGMT-promoter methylated compared to non-methylated tumors. Mean OS in the non-methylated group was 16.9 months (Std 1.89, 95% CI 13.2–20.6) compared to 36.1 (Std 6.57, 95% CI 23.2–49.0) in the methylated group (Table 6). PFS was 6.1 months (Std 0.66, 95% CI 4.8–7.4) and 14.2 months (Std 3.87, 95% CI 6.6–21.7) respectively (Table 7).

**Table 6:** Overall survival in months between O6-methylguanine-DNA methyltransferase (MGMT) status and between larger/lower than median region of interest (ROI) volume in all ROIs segmented through deep learning methods.

Overall survival based on MGMT mean, Std and 95% CI shown in months					
	*p*-value	Comparison	Mean	Std	95% CI
MGMT methylated vs. non-methylated	*p* < 0.01	MGMT met	36.1	6.57	23.2–49.0
		Non-met	16.9	1.89	13.2–20.6

Overall survival comparison in patients larger (larger) or lower/equal (smaller) than median ROI volume shown in months						
Segmentation method	ROI	*p*-value	Comparison	Mean	Std	95% CI
DeepBraTumIA	CE tumor volume	*p* < 0.01	Smaller	37.7	6.90	24.2–51.2
			Larger	16.0	1.59	12.9–19.1
	Tumor necrosis volume	*p* = 0.25	Smaller	29.3	5.49	18.6–40.1
			Larger	22.4	3.69	15.2–29.6
	Total tumor volume	*p* = 0.30	Smaller	29.3	5.49	18.5–40.0
			Larger	22.5	3.71	15.2–29.7
	Peritumoral edema volume	*p* = 0.75	Smaller	27.3	5.86	15.8–38.8
			Larger	25.1	4.10	17.1–33.1
	Total-tumor/edema ratio	*p* = 0.02	Smaller	31.8	5.39	21.2–42.3
			Larger	19.9	3.66	12.8–27.1
Raidionics	CE tumor volume	*p* < 0.01	Smaller	36.2	6.74	23.0–49.4
			Larger	16.8	1.57	13.7–19.9
	Tumor necrosis volume	*p* = 0.04	Smaller	31.5	5.59	20.6–42.5
			Larger	20.2	3.46	13.5–27.0
	Total tumor volume	*p* = 0.01	Smaller	35.1	6.60	22.2–48.0
			Larger	17.3	1.69	14.0–20.6
	Peritumoral edema volume	*p* = 0.35	Smaller	25.0	5.22	14.8–35.3
			Larger	25.6	3.68	18.4–32.8
	Total-tumor/edema ratio	*p* < 0.01	Smaller	35.3	6.20	23.1–47.4
			Larger	19.7	3.09	11.7–23.8

Std, standard deviation; 95% CI, 95% confidence interval; Met., methylated.

**Table 7:** Progression free survival in months between O6-methylguanine-DNA methyltransferase (MGMT) status and between larger/lower than median region of interest (ROI) volume in all ROIs segmented through deep learning methods.

Progression free survival based on MGMT mean, Std and 95% CI shown in months					
	*p*-value	Comparison	Mean	Std	95% CI
MGMT methylated vs. non-methylated	*p* = 0.03	MGMT-met	14.2	3.87	6.6–21.7
		Non-met	6.2	0.66	4.8–7.5

Progression free survival comparison in patients larger (larger) or lower/equal (smaller) than median ROI volume shown in months						
Segmentation method	ROI	*p*-value	Comparison	Mean	Std	95% CI
DeepBraTumIA	CE tumor volume	*p* = 0.01	Smaller	14.3	4.13	6.2–22.4
			Larger	6.4	0.93	4.6–8.2
	Tumor necrosis volume	*p* = 0.60	Smaller	9.6	2.06	5.5–13.6
			Larger	10.0	3.07	4.0–16.0
	Total tumor volume	*p* = 0.33	Smaller	9.8	2.02	5.9–13.8
			Larger	9.9	3.17	3.6–16.1
	Peritumoral edema volume	*p* = 0.37	Smaller	10.0	3.46	3.2–16.7
			Larger	10.0	1.98	6.1–13.8
	Total-tumor/edema ratio	*p* = 0.44	Smaller	9.9	2.22	5.6–14.3
			Larger	9.6	2.87	4.0–15.3
Raidionics	CE tumor volume	*p* = 0.07	Smaller	13.7	4.05	5.7–21.6
			Larger	6.8	0.94	5.0–8.6
	Tumor necrosis volume	*p* = 0.69	Smaller	9.6	2.08	5.5–13.6
			Larger	10.2	3.10	4.1–16.2
	Total tumor volume	*p* = 0.04	Smaller	13.6	3.87	6.0–21.2
			Larger	6.7	1.04	4.7–8.7
	Peritumoral edema volume	*p* = 0.26	Smaller	8.0	1.75	4.6–11.5
			Larger	11.9	3.6	4.9–18.9
	Total-tumor/edema ratio	*p* = 0.03	Smaller	13.8	3.92	6.1–21.5
			Larger	6.6	1.07	4.5–8.7

Std, standard deviation; 95% CI, 95% confidence interval; Met., methylated.

Analyzing volumes from DeepBraTumIA we found significantly higher OS in lower volumes of contrast enhancing tumor (*p* < 0.01) (lower volume mean 37.7 months, Std 6.90, 95% CI 21.2–51.2 vs. higher volume mean 16.0 months, Std 1.59, 95% CI 12.9–19.1) and in lower total-tumor/edema ratio (*p* = 0.02) (lower ratio mean 31.8 months, Std 5.39, 95% CI 21.2–42.3 vs. higher ratio mean 19.9 months, Std 3.66, 95% CI 12.8–27.1) (Table 6). PFS was significantly higher in lower volumes of contrast enhancing tumor (*p* = 0.01) (lower volume mean 14.3 months, Std 4.13, 95% CI 6.2–22.4 vs. higher volume mean 6.4 months, Std 0.93, 95% CI 4.6–8.2) (Table 7). The remaining volumes showed no significant differences.

Analyzing volumes from Raidionics we found significantly higher OS in lower contrast enhancing volume (*p* < 0.01) (lower volume mean 36.2 months, Std 6.74, 95% CI 23.0–49.4 vs. higher volume mean 16.8 months, Std 1.57, 95% CI 13.7–19.9), lower necrosis volume (*p* = 0.04) (lower volume mean 31.5 months, Std 5.59, 95% CI 20.6–42.5 vs. higher volume mean 20.2 months, Std 3.46, 95% CI 13.5–27.0), lower total tumor volume (*p* = 0.01) (lower volume mean 35.1 months, Std 6.60, 95% CI 22.2–48.0 vs. higher volume mean 17.3 months, Std 1.69, 95% CI 14.0–20.6) and lower total-tumor/edema ratio (*p* < 0.01) (lower ratio mean 35.3 months, Std 6.20, 95% CI 23.1–47.4 vs. higher ratio mean 17.7 months, Std 3.09, 95% CI 11.7–23.8) (Table 6). Significantly higher PFS was observed in lower total tumor volume (*p* = 0.04) (lower volume mean 13.6 months, Std 3.87, 95% CI 6.0–21.2 vs. higher volume mean 6.7 months, Std 1.04, 95% CI 4.7–8.7) and lower total-tumor/edema ratio (*p* = 0.03) (lower ratio mean 13.8 months, Std 3.92, 95% CI 6.1–21.5 vs. higher ratio mean 6.6 months, Std 1.07, 95% CI 4.5–8.7) (Table 7). The remaining volumes showed no significant differences.

#### 3.4.2 GBM-subgroup

OS and PFS were shown to be significantly higher (*p* < 0.01 and *p* = 0.04 respectively) in MGMT-promoter methylated compared to non-methylated tumors. Mean OS in the non-methylated group was 21.9 months (Std 1.75, 95% CI 12.5–19.4) compared to 30.0 (Std 6.29, 95% CI 17.7–42.3) in the methylated group (Table 8). PFS was 5.7 months (Std 0.63, 95% CI 4.5–6.9) and 10.2 months (Std 2.38, 95% CI 5.6–14.9) respectively (Table 9).

**Table 8:** Overall survival in months in glioblastoma (GBM) subgroup between O6-methylguanine-DNA methyltransferase (MGMT) status and between larger/lower than median region of interest (ROI) volume in all ROIs segmented through deep learning methods.

Overall survival based on MGMT mean, Std and 95% CI shown in months					
	*p*-value	Comparison	Mean	Std	95% CI
MGMT methylated vs. non-methylated	*p* = 0.01	MGMT-met	30.0	6.29	17.7–42.3
		Non-met	21.9	1.75	12.5–19.4

Overall survival comparison in patients larger (larger) or lower/equal (smaller) than median ROI volume shown in months						
Segmentation method	ROI	*p*-value	Comparison	Mean	Std	95% CI
DeepBraTumIA	CE tumor volume	*p* < 0.01	Smaller	29.4	5.88	17.9–40.9
			Larger	15.0	1.52	12.0–18.0
	Tumor necrosis volume	*p* = 0.03	Smaller	28.2	5.82	16.8–39.6
			Larger	15.8	1.39	13.1–18.6
	Total tumor volume	*p* = 0.02	Smaller	30.0	5.96	17.3–40.7
			Larger	15.5	1.51	12.5–18.5
	Peritumoral edema volume	*p* = 0.39	Smaller	20.5	4.66	11.4–29.6
			Larger	23.4	3.95	15.6–31.1
	Total-tumor/edema ratio	*p* < 0.01	Smaller	30.9	5.73	19.7–42.1
			Larger	13.3	1.40	10.6–16.1
Raidionics	CE tumor volume	*p* < 0.05	Smaller	28.0	5.86	16.6–39.5
			Larger	16.1	1.49	13.2–19.0
	Tumor necrosis volume	*p* < 0.01	Smaller	28.5	5.40	17.9–39.1
			Larger	14.7	1.32	12.1–17.3
	Total tumor volume	*p* = 0.02	Smaller	28.4	5.80	17.0–39.8
			Larger	15.7	1.51	12.7–18.6
	Peritumoral edema volume	*p* = 0.70	Smaller	23.3	5.36	12.8–33.8
			Larger	20.0	1.84	16.4–23.7
	Total-tumor/edema ratio	*p* < 0.01	Smaller	31.4	5.6	20.4–42.4
			Larger	12.6	1.26	10.2–15.1

Std, standard deviation; 95% CI, 95% confidence interval; Met., methylated.

**Table 9:** Progression free survival in months in glioblastoma (GBM) subgroup between O6-methylguanine-DNA methyltransferase (MGMT) status and between larger/lower than median region of interest (ROI) volume in all ROIs segmented through deep learning methods.

Progression free survival based on MGMT Mean, Std and 95% CI shown in months					
	*p*-value	Comparison	Mean	Std	95% CI
MGMT methylated vs. non-methylated	*p* = 0.04	MGMT-met	10.2	2.38	5.6–14.9
		Non-met	5.7	0.63	4.5–6.9

Progression free survival comparison in patients larger (Larger) or lower/equal (Smaller) than median ROI volume shown in months.						
Segmentation method	ROI	*p*-value	Comparison	Mean	Std	95% CI
DeepBraTumIA	CE tumor volume	*p* = 0.01	Smaller	9.7	2.24	5.3–14.1
			Larger	5.8	0.61	4.6–7.0
	Tumor necrosis volume	*p* = 0.03	Smaller	9.8	2.16	5.6–14.0
			Larger	5.5	0.54	4.5–6.6
	Total tumor volume	*p* = 0.02	Smaller	10.2	2.19	5.9–14.5
			Larger	5.3	0.56	4.2–6.4
	Peritumoral edema volume	*p* = 0.39	Smaller	6.3	0.82	4.7–7.9
			Larger	8.8	1.98	4.9–12.6
	Total-tumor/edema ratio	*p* < 0.01	Smaller	10.0	2.35	5.4–14.6
			Larger	5.7	0.69	4.4–7.0
Raidionics	CE tumor volume	*p* = 0.17	Smaller	9.3	2.22	4.9–13.6
			Larger	6.2	0.62	4.96–7.4
	Tumor necrosis volume	*p* = 0.08	Smaller	9.4	2.09	5.3–13.5
			Larger	5.8	0.54	4.7–6.8
	Total tumor volume	*p* = 0.02	Smaller	9.8	2.16	5.6–14.0
			Larger	5.5	0.55	4.5–6.6
	Peritumoral edema volume	*p* = 0.70	Smaller	8.4	1.88	4.7–12.1
			Larger	6.6	0.86	4.9–8.3
	Total-tumor/edema ratio	*p* < 0.01	Smaller	10.2	2.31	5.7–14.7
			Larger	5.5	0.70	4.1–6.8

Std, standard deviation; 95% CI, 95% confidence interval; Met., methylated.

Analyzing volumes from DeepBraTumIA we found significantly higher OS in lower volumes of contrast enhancing tumor (*p* < 0.01) (lower volume mean 29.4 months, Std 5.88, 95% CI 17.9–40.9 vs. higher volume mean 15.0 months, Std 1.52, 95% CI 12.0–18.0), in lower tumor necrosis (*p* = 0.03) (lower volume mean 28.2 months, Std 5.82, 95% CI 16.8–39.6 vs. higher volume mean 15.8 months, Std 1.39, 95% CI 13.1–18.6), in lower total tumor volume (*p* = 0.02) (lower volume mean 30.0 months, Std 5.96, 95% CI 17.3–40.7 vs. higher volume mean 15.5 months, Std 1.51, 95% CI 12.5–18.5) and in lower total-tumor/edema ratio (*p* < 0.01) (lower ratio mean 30.9 months, Std 5.73, 95% CI 19.7–42.1 vs. higher ratio mean 13.3 months, Std 1.40, 95% CI 10.6–16.1) (Table 8). PFS was significantly higher in lower volumes of contrast enhancing tumor (*p* = 0.01) (lower volume mean 9.7 months, Std 2.24, 95% CI 5.3–14.1 vs. higher volume mean 5.8 months, Std 0.61, 95% CI 4.6–7.0), in lower tumor necrosis (*p* = 0.03) (lower volume mean 9.8 months, Std 2.16, 95% CI 5.6–14.0 vs. higher volume mean 5.5 months, Std 0.54, 95% CI 4.5–6.6), in lower total tumor volume (*p* = 0.02) (lower volume mean 10.2 months, Std 2.19, 95% CI 5.9–14.5 vs. higher volume mean 5.3 months, Std 0.56, 95% CI 4.2–6.4) and in lower total-tumor/edema ratio (*p* < 0.01) (lower ratio mean 10.0 months, Std 2.35, 95% CI 5.4–14.6 vs. higher ratio mean 5.7 months, Std 0.69, 95&CI 4.4–7.0) (Table 9). The remaining volumes showed no significant differences.

Analyzing volumes from Raidionics we found significantly higher OS in lower contrast enhancing volume (*p* < 0.05) (lower volume mean 28.0 months, Std 5.86, 95% CI 16.6–39.5 vs. higher volume mean 16.1 months, Std 1.49, 95% CI 13.2–19.0), lower necrosis volume (*p* < 0.01) (lower volume mean 28.5 months, Std 5.40, 95% CI 17.9–39.1 vs. higher volume mean 14.7 months, Std 1.32, 95% CI 12.1–17.3), lower total tumor volume (*p* = 0.02) (lower volume mean 28.4 months, Std 5.80, 95% CI 17.0–39.8 vs. higher volume mean 15.7 months, Std 1.51, 95% CI 12.7–18.6) and lower total-tumor/edema ratio (*p* < 0.01) (lower ratio mean 31.4 months, Std 5.6, 95% CI 20.4–42.4 vs. higher ratio mean 12.6 months, Std 1.26, 95% CI 10.2–15.1) (Table 8). Significantly higher PFS was observed in lower total tumor volume (*p* = 0.02) (lower volume mean 9.8 months, Std 2.16, 95% CI 5.6–14.0 vs. higher volume mean 5.5 months, Std 0.55, 95% CI 4.5–6.6) and lower total-tumor/edema ratio (*p* < 0.01) (lower ratio mean 10.2 months, Std 2.31, 95% CI 5.7–14.7 vs. higher ratio mean 5.5 months, Std 0.70, 95% CI 4.1–6.8) (Table 9). The remaining volumes showed no significant differences.

## 4 Discussion

The ability to accurately predict MGMT-promoter methylation preoperatively could play a vital role in the treatment of HGG. Using volumes gathered by segmenting ROIs, which is more effectively done using DL-based segmentation models, might aid in this distinction. Therefore, this study examined contrast enhancing tumor, central necrosis and peritumoral edema volumes using both automatic and manual segmentation methods, in HGG patients with and without MGMT promotor methylation.

Our data show that larger peritumoral edema volumes, segmented using DeepBraTumIA, positively predicts MGMT promoter methylation. These findings could be in line with Li et al. (2011) who found that low expression of MGMT was associated with presence of more severe edema. However, they did not explore any volumetric correlations beyond visual inspection. Additionally other studies contradict our findings, with one study not finding any significant differences between methylated and non-methylated groups (Kanas et al., 2017) and others having identified MGMT promotor methylation in tumors with lower edema volume (Suh et al., 2018; Kanas et al., 2017; Koska and Koska, 2025).

Although we did find significant group differences in the peritumoral edema ROI with DeepBraTumIA, the ROC analysis (AUC = 0.638) indicates limited clinical utility. These findings are consistent with previous research which, despite showing lower volume of peritumoral edema in MGMT-methylated tumors, also struggles with achieving high predictive potential on models created through a single parameter (Kanas et al., 2017; Koska and Koska, 2025). These discoveries indicate that the use of peritumoral edema volume alone as a predictive MRI feature is not feasible. This conclusion is further supported by our multivariate analysis, where the peritumoral edema ROI failed to remain a significant predictor when tested against other clinical covariates.

It is important to note that peritumoral edema represents a downstream effect of a tumor, and may not be directly linked to expression of MGMT. It can be speculated that factors such as vascular endothelial growth factor (VEGF) and matrix metalloproteinases (MMPs), expressed by the tumor, may play a more prominent role in mediating peritumoral edema trough their effect on the blood brain barrier (BBB) (Ohmura et al., 2023). Previous studies indicate that expression of VEGF within the brain may increase BBB leakage and the formation of abnormal tumor vessels, causing increased localized edema burden (Ohmura et al., 2023; Zhang et al., 2000). Furthermore, extensive expression of MMPs causes destruction of the basal membrane of the BBB, resulting in edema (Ohmura et al., 2023; Zhang et al., 2019). Consequently, the limited predictive ability of peritumoral edema in our study may reflect that such secondary pathophysiological processes, rather than MGMT promoter methylation itself, exert the dominant influence on edema formation.

Additionally, tumor edema is influenced by symptomatic intake of corticosteroids. Non-MGMT-methylated tumors may have more aggressive clinical features and thus a higher proportion of steroid use pre-operatively. Since most patients in our cohort had either recently initiated corticosteroid therapy or were on a low to no dose at the time of imaging, we expect the impact of such treatment to be minimal on the peritumoral edema. Additionally, we observed no significant difference in mean dosage of betamethasone between patients stratified by MGMT status (Table 1). Our multivariate analysis also showed no association between corticosteroid dosage and MGMT in the tested prediction models. This suggests that corticosteroids did not act as a confounder in our predictive model including the tumor/edema ratio.

Some studies have found a lower survival rate in patients with larger volumes of peritumoral edema (Carrillo et al., 2012; Pope et al., 2005; Hammoud et al., 1996). The lower survival rate is theorized to be related to the increased mass effect of the peritumoral edema on the surrounding tissue (Carlson et al., 2007). As previously mentioned, peritumoral edema could be a consequence of increased expression of VEGF from the HGG. This could potentially indicate a more hypoxic and aggressive tumor (Carlson et al., 2007). Lower survival rates are also reported in patients with VEGF-expressing HGG and high peritumoral edema volumes, whereas VEGF-positive tumors with low or no peritumoral edema had no significant impact on survival (Carlson et al., 2007), supporting the notion that larger peritumoral edema is found within more aggressive tumors. Additionally, pro-tumor associated gene expressions, such as p53 suppression, have been found within peritumoral edema lowering the delay between surgery and tumor recurrence (Luo et al., 2021). However, our findings are contradictory to these findings, suggesting no difference in OS or PFS based on edema burden. Furthermore, we can conclude that MGMT-promoter methylated tumors offer significantly higher OS compared to non-methylated. Considering our findings that methylated tumors express larger volumes of peritumoral edema we theorize that edema burden might not be as impactful on OS compared to the contrast-enhancing tumor volume and total tumor/edema ratio (Carrillo et al., 2012).

As mentioned previously our results are contradictory to some previous studies, primarily those which identified MGMT methylation in tumors with lower amount of peritumoral edema volume (Suh et al., 2018; Kanas et al., 2017; Koska and Koska, 2025). It is possible that patients in our cohort expressing MGMT promoter methylation may show up during later stages of their disease, compared to non-methylated, if the tumor is less aggressive. Furthermore, previous studies have demonstrated that tumors with higher degree of MGMT methylation, measured with pyrosequencing, exhibit a larger volumetric peritumoral edema reduction as response to eventual treatment, and prolonged survival (Hosoya et al., 2022). This agrees with our results, which showed a longer OS in patients with MGMT methylated tumors, despite having larger preoperative peritumoral edema. It is important to note that the MGMT-methylated group included a significantly higher proportion of grade 4 astrocytoma patients compared to the non-methylated group. However, we see similar results in OS and PFS in the GBM subgroup analysis when looking at MGMT methylation. Furthermore, it’s important to note that there is currently no robust imaging-based method to differentiate between GBM and astrocytoma grade 4 pre-operatively when assessing HGG. As a result, it would be of limited clinical value to further assess the effect of the skewed group composition. Additionally, the inclusion of both subtypes in the overall cohort reflects the clinical reality faced at diagnosis while the OS and PFS findings indicate a correlation between the mixed group and GBM subgroup in relation to MGMT-methylation (significantly higher in both) suggesting that the observed increased survival may be driven by MGMT status rather than group composition.

The largest changes to OS and PFS between the GBM subgroup and the mixed cohort were observed in tumor necrosis volume, total tumor volume, and total-tumor/edema ratio segmented by DeepBraTumIA. In the GBM-only analysis, all of these showed significantly longer OS or PFS in patients with lower volumes/ratio, whereas no such associations were seen when astrocytomas were included. However, volumes segmented by Raidionics did not show such a change in results in the subgroup. This pattern suggests that the differences observed are a consequence of DeepBraTumIA ability to segment astrocytomas in the mixed cohort, rather than reflecting true OS and PFS differences between the mixed cohort and the subgroup.

We also found the total-tumor/edema ratio to be higher in non-MGMT promoter methylated tumors. A higher ratio is derived from a larger total tumor or smaller peritumoral edema volume, which aligns with our findings showing larger peritumoral edema volume in MGMT-promoter methylated tumors. Using segmentation from both DL-models significant differences and improved predictive capabilities were found in total-tumor/edema ratio which could indicate that the combination of multiple ROIs could have a larger applicability and be less sensitive to specific segmentation methods, compared to individual ROIs. This is in line with previous studies showing higher AUC values when combining multiple metrics (Li et al., 2024). Furthermore, our multivariate analysis indicates that lower ratio was predictive of MGMTpm methylation and that the predictive findings are truly related to regional variations seen on MRI instead of a consequence of other covariates. Ratios segmented by both DL-models were the only significant factor in the logistic regression models when tested against our clinical covariates. Such results were not present when modeling using peritumoral edema segmented by DeepBraTumIA, strengthening the hypothesis that a singular ROI is not sufficient in capturing the complexity of HGG and predicting MGMT-methylation. Despite all these findings, the ratio’s predictive utility is limited by its low AUC in ROC analysis while improving prediction over baseline in multivariate regression.

We observed improved predictive ability in both total-tumor/edema ratios when analyzing the GBM subgroup compared to the mixed cohort, albite only by a slight margin. Despite the prediction not being clinically applicable, the implication of these findings is important to note. As previously mentioned, no robust pre-operative imaging method currently exists to distinguish HGG gliomas from each other in pre-operative imaging. As such, knowing that we have similar ability to predict MGMT methylation in both a mixed cohort and GBM subgroup indicates that further studies using cohorts that mirror this clinical reality are warranted.

Whereas we report a higher total-tumor/edema ratio to be predictive of non MGMT-methylation, another study reports the opposite (Kanas et al., 2017) while other studies found no correlation between MRI features and MGMT promoter methylation (Kim et al., 2022; Mikkelsen et al., 2020). The discrepancy could indicate other factors in play which could impact peritumoral edema.

We speculate that one such factor could be the immune response surrounding the tumor. Previous studies suggest that MGMT status may modulate and impact the local immune response to the tumor (Kushihara et al., 2024). It is suggested that higher expressions of MGMT may increase CD8 T-cells abundance (Kushihara et al., 2024). This response may in turn change the microenvironment surrounding the tumor in a way which promotes a heightened inflammatory response and subsequently increased peritumoral edema (Engelhorn et al., 2009). This is further complicated by the heterogeneous expression of MGMT within the tumor, as each subpopulation within the tumor may create distinct unique microenvironment due to different levels of immune cell recruitment (Singh et al., 2025). Collectively, these factors highlight the complexity of MGMT prediction in HGG based on a single factor approach. They also raise the possibility that varying degrees of homogeneity and expression of MGMT may impact prediction even within non methylated MGMT tumors, which could be explored in future studies. Some studies have shown that factors such as apparent diffusion coefficient (ADC) (Rundle-Thiele et al., 2015; Kanazawa et al., 2019) and textural differences of the images (Drabycz et al., 2010) could instead be used as a basis for an MGMT-methylation prediction model. Furthermore, amide proton transfer weighted imaging has recently also shown promising results in predicting MGMT in GBM and grade 4 astrocytoma (Durmo et al., 2025). Given the complex nature of both the brain and HGG, we believe that a singular-factor approach may be insufficient for predicting MGMT methylation. Our multivariate analysis confirms this theory. While our models did become statistically significant while accounting for co-variates, the overall predictive ability remained limited with a singular ROI. A more holistic study design, incorporating multiple factors and their relation to each other, could be a better approach to find factors which predict MGMT-promotor methylation. Thus, future studies should take multiple factors into consideration, looking for a linked correlation between the overall tumor image and MGMT status, instead of focusing on a singular ROI.

Due to the limited predictive performance of our models, we chose not to perform PFS or OS analyses stratified by predicted MGMT status. Given the modest AUC values observed for our ROIs, such analyses would not meaningfully reflect the prognostic impact of MGMT methylation but rather the effects of the volumetric features themselves on survival. The effect of higher and lower ROI volumes on PFS and OS is an association we already demonstrated. As a result, we concluded that additional survival analyses based on predicted MGMT status are unlikely to offer added clinical insights. We observed, when comparing DL-model segmentation to manual segmentation, that Raidionics differ in fewer ROIs than DeepBraTumIA. This discrepancy may be due to differences in model architecture or potential biases in Raidionics’ training data that better align with our dataset. However, these findings do not necessarily indicate that Raidionics is a superior segmentation model, only that it corresponds to a higher degree with our specific manual segmentation. Given the well-documented inter-observer variability among experienced radiologists (Erasmus et al., 2003; Zhao et al., 2013; Barboriak et al., 2019), manual segmentation itself shows considerable variation. Rather than comparing DL models solely to human segmentation, their performance should be assessed based on task-specific accuracy. Thus, their agreeability in the ability to predict MGMT-methylation suggests that they are equally suited for that task.

Analysis of the Dice coefficients reveals that tumor necrosis is the most inconsistent ROI across all segmentation methods. No comparison had greater than 73% overlap, in contrast with the remaining ROIs which had a range of 82–91%. These results indicate a large discrepancy in tumor necrosis definition among the segmentation methods. For the manual segmentation the tumor necrosis was defined as a hypointense area, surrounded by contrast enhancing tumor on the T1-GD images. Visual observation reveals that in the DL models’ segmentations, tumor necrosis may be defined as hypointense regions adjacent to contrast enhancing tumor tissue, resulting in the labeling of necrosis outside of the contrast enhancing region. This can be seen when comparing the performed DL segmentations in two different patients (Figure 3). These findings highlight the need for a more robust and standardized segmentation criteria for tumor necrosis. The DL-models rely on their segmentation of training data that incorporates true labels from experienced radiologists. The observed discrepancy between segmentation methods likely reflects an underlying bias and variability within the training data. This is further supported by the results of the ICC, showing excellent inter-observer agreement for contrast-enhancing tumor and edema ROIs, while at best having moderate agreement within the necrosis ROI. Previous studies have also identified similar limitations in using DL-models on segmentation (Bonada et al., 2024). Furthermore, as tumor necrosis volume is the smallest ROI across all segmentation methods, even minor differences in labeling definitions could result in large impact on the dice-coefficient. DeepBraTumIA, judging by volume, seems to be more likely to over segment tumor necrosis, compared to the other segmentation methods.

Such findings are likely to impact on our ability to reliably predict MGMT promoter methylation within the tumor necrosis ROI. Even if significant group differences were observed, clinical applicability would remain limited, as high interobserver variability would translate to high variability in prediction. However, due to the definition used for other ROIs in our study, especially total tumor volume and in turn total-tumor/edema ratio, we expect that the downstream effect on them to be minimal.

Our study has some limitations. Firstly, our study employs a single center study design with a cohort of 70 patients, limiting statistical power and generalizability. For future studies a wider dataset from multiple centers is recommended to enhance the generalizability of the results and improve statistical power. Secondly, the manual segmentations were in large performed by a single assessor. It could have been performed by multiple readers independently, to account for inter-observer variance and improve the comparison between the manual and DL segmentations. However, the manual segmentation performed by EZ was reviewed by both an experienced researcher (TS) and a neuroradiologist with more than 30 years of experience (PS). Additionally, the manual segmentation in our study was only performed in a subgroup of patients as an initial validation of the DL models’ performance, as the main aim was to evaluate imaging features in the predictive ability of MGMT status. Lastly, due to the complexity of segmentation and deep learning, a large dataset, using labeling and segmenting from different radiologists, would be recommended to accurately compare DL-models to each other. Recent studies have highlighted this methodology by using large multi-institutional datasets to develop and validate a post-operative DL-model against other tools, such as Raidionics (Cepeda et al., 2024).

## 5 Conclusion

This study demonstrated significant differences between MGMT promoter methylated and non-methylated HGG in the peritumoral edema volume and total-tumor/edema ratio. Total-tumor/edema ratio seems to be the single most predictive factor for MGMT-status using multivariate analysis. However, the diagnostic performance of both peritumoral edema and ratio were limited. OS and PFS were longer in the MGMT-promoter methylated group. Volumes obtained from both DL-models were both equally suited for predicting MGMT status, highlighting the potential use of such models clinically as a tool to help radiologists.

## Data Availability

The raw data supporting the conclusions of this article will be made available by the authors, without undue reservation.

## Glossary

ADC: Apparent diffusion coefficient
AUC: Area under curve
BBB: Blood brain barrier
BraTS: Brain tumor segmentation
DL: Deep learning
GBM: Glioblastoma
GTR: Gross total resection
HD-BET: HD Brain Extraction Tool
HGG: High grade glioma
ICC: Intra-class correlation coefficient
IDH: Isocitrate dehydrogenase
MGMT: O6-methylguanine-DNA methyltransferase
MMP: Matrix metalloproteinase
MPRAGE: Magnetization prepared rapid gradient echo
MRI: Magnetic resonance imaging
OS: Overall survival
PCR: Polymerase chain reaction
PFS: Progression free survival
ROC: Receiver operation characteristics
ROI: Region of interest
RT: Radiotherapy
T1-GD: T1 gadolinium contrast
T2-FLAIR: T2-weighted fluid-attenuated inversion recovery
TE: Echo time
TI: Inversion time
TMZ: Temozolomide
TR: Repetition time
TSE: Turbo spin echo
VEGF: Vascular endothelial growth factor
